# Ethnic density, urbanicity and psychosis risk for migrant groups – A population cohort study

**DOI:** 10.1016/j.schres.2017.03.032

**Published:** 2017-12

**Authors:** Peter Schofield, Malene Thygesen, Jay Das-Munshi, Laia Becares, Elizabeth Cantor-Graae, Carsten Pedersen, Esben Agerbo

**Affiliations:** a Division of Health & Social Care Research, Faculty of Life Sciences & Medicine, King's College London, London, United Kingdom; b Health Service & Population Research Department, Institute of Psychiatry, Psychology & Neuroscience, King's College London, London, United Kingdom; c Lundbeck Foundation Initiative for Integrative Psychiatric Research, iPSYCH, Aarhus, Denmark; d National Centre for Register-Based Research, Aarhus University, Aarhus, Denmark; e CIRRAU - Centre for Integrated Register-based Research at Aarhus University, Aarhus, Denmark; f Centre on Dynamics of Ethnicity, The University of Manchester, Manchester, United Kingdom; g Social Medicine and Global Health, Lund University, Lund, Sweden

**Keywords:** Etiology, Social determinants, Ethnicity, Psychosis

## Abstract

**Background:**

Rates of psychotic disorder are raised for many migrant groups. Understanding the role played by the social context in which they live may help explain why. This study investigates the effect of both neighbourhood ethnic density and urbanicity on the incidence of non-affective psychosis for migrant groups.

**Method:**

Population based cohort of all those born 1965 or later followed from their 15th birthday (2,224,464 people) to 1st July 2013 (37,335,812 person years). Neighbourhood exposures were measured at age 15.

**Results:**

For all groups incidence of non-affective psychosis was greater in lower ethnic density neighbourhoods. For migrants of African origin there was a 1.94-fold increase (95% CI, 1.17–3.23) comparing lowest and highest density quintiles; with similar effects for migrants from Europe (excluding Scandinavia): incidence rate ratio (IRR) 1.99 (95% CI, 1.56–2.54); Asia: IRR 1.63 (95% CI, 1.02–2.59); and the Middle East: IRR 1.68 (95% CI, 1.19–2.38). This initial analysis found no evidence for an urbanicity effect for migrant groups. Adjusting for ethnic density revealed a positive association between level of urbanicity and psychosis for two groups, with a statistically significant linear trend (average effect of a one quintile increase) for migrants from Europe: IRR 1.09 (95% CI, 1.02–1.16) and the Middle East: IRR 1.12 (95% CI, 1.01–1.23).

**Conclusions:**

In this first nationwide population-based study of ethnic density, urbanicity and psychosis we show that lower ethnic density is associated with increased incidence of non-affective psychosis for different migrant groups; masking urban/rural differences in psychosis for some groups.

## 1 Introduction

An elevated risk of psychosis among some migrant groups is well documented and, in some instances, estimated to be greater than most other risk factors with the exception of family history of psychosis (Bourque et al., 2011, Cantor-Graae and Pedersen, 2013, Cantor-Graae and Selten, 2005, McGrath et al., 2004). It is unlikely that this is due to selective migration (Pedersen et al., 2011, van der Ven et al., 2015) and international comparison studies have failed to show any corresponding increased incidence in the country of origin (Bhugra et al., 1996, Jablensky et al., 1992). Reviews show elevated rates persist from first to second generation migrants pointing to the relevance of the social context post-migration (Bourque et al., 2011, Cantor-Graae and Selten, 2005).

An ethnic density effect has been observed where psychosis incidence is reduced for members of minority ethnic groups who live in areas where their ethnic group is well represented (Becares et al., 2009, Boydell et al., 2001, Kirkbride et al., 2007b, Veling et al., 2008). This provides arguably the most compelling evidence for the social environment having an important aetiological role (March et al., 2008). However, we can only draw limited conclusions from studies to date as the causal pathway is typically obscured, with exposure (neighbourhood) determined either near to or at the point of diagnosis. Only one study has looked at prior exposure (Zammit et al., 2010) showing higher rates of psychotic illness for foreign born pupils in schools with fewer foreign born pupils, but this was not able to distinguish between ethnic groups.

A related issue is urbanicity, where urban birth and upbringing are repeatedly associated with increased risk of psychosis (Krabbendam and van Os, 2005, Pedersen, 2006, Vassos et al., 2012). Only one previous study has looked at this for migrant groups finding no relation (Cantor-Graae and Pedersen, 2007). The authors speculate this may be because higher ethnic density in urban areas has a protective effect but, to date, no study has examined how these two potentially opposing forces act. There is evidence that individual-level socio-economic background is also relevant (Kirkbride et al., 2014, Kirkbride et al., 2007a, Morgan et al., 2008, Werner et al., 2007). However, most studies cannot distinguish this from the effects of early or prodromal illness and, it is argued, more attention should be paid to parental socio-economic background (Cantor-Graae and Selten, 2005, Morgan et al., 2008).

Ideally studies would therefore follow subjects prospectively, charting neighbourhood exposure and parental background in childhood, and be on a scale that can differentiate between migrant groups. This is the first study to do this, using a nationwide population-based sample to examine the joint effects of neighbourhood ethnic density and urbanicity on risk of non-affective psychosis.

## 2 Method

### 2.1 Data Source

Since 1968 all those resident in Denmark have a unique personal identification number allowing data to be linked at an individual level across population registers. We used the Danish Civil Registration System dataset which includes demographic details and links to parents as well as continuous updates on place of residence and vital status (Pedersen et al., 2006).

### 2.2 Cohort

We followed all those born between 1st January 1965 and 31 December 1997 and living in Denmark on their 15th birthday until they either died, migrated, were diagnosed with a non-affective psychotic illness or 1st of July 2013 (whichever came first).

### 2.3 Outcome

The cohort, and their parents, were linked to the Danish Psychiatric Central Register (Munk-Jørgensen and Mortensen, 1997) which covers all psychiatric in-patient admissions and, from 1995, out-patient visits. We defined non-affective psychosis as ICD-10 codes F20-F29 and their ICD-8 equivalents (ICD-8295.× 9, 296.89, 297.× 9, 298.29–298.99, 299.04, 299.05, 299.09, 301.83) following the method used previously (Pedersen et al., 2014). This was based on clinical diagnoses assigned at discharge, shown to have good diagnostic validity (Jakobsen et al., 2005, Uggerby et al., 2013). Date of onset was defined as the first day of first contact with this diagnosis, and we excluded anyone with a diagnosis prior to their 15th birthday.

### 2.4 Definition of migrant group

We defined members of a migrant group as anyone born outside of Denmark (first generation) or born in Denmark but with both parents born outside Denmark (second generation). We used the country of origin of both parents as this has been shown to be most clearly related to psychosis risk, and categorised country of origin in the same way as previously (Cantor-Graae et al., 2003, Cantor-Graae and Pedersen, 2007). We retained the four largest groups: migrants from Africa, Europe (other than Scandinavia), Asia (Indian sub-continent, China and South East Asia) and the Middle East. Country of origin was missing for a total of 31,748 (1.4%) either because their place of birth was missing or they were born in Denmark and this was missing for either parent. We excluded a further 94,489 (4.4%) born in Denmark with parents born in different regions and therefore not easily classified.

### 2.5 Neighbourhood effects

Neighbourhood units were based on Danish parishes which vary considerably in size hindering model convergence. For small parishes we therefore combined adjacent units to arrive at an optimum size, using AZtool, the algorithm devised to create UK census area units (Cockings et al., 2011, Martin, 2003). We set the algorithm to aim for an optimum parish size of 3000 inhabitants with no units < 200, collapsing 2114 parishes into 1135 units. We also split very large parishes (over 6500) into two, randomly assigning parish members into either unit, giving a final total of 1167 parish units (median size 3564). These were then used to determine the neighbourhood social context based on all residents in the parish in any one year. For each parish and migrant group (defined above) ethnic density was defined as the proportion from that group in the parish in the year the cohort member was 15, divided into quintiles. We chose neighbourhood at age 15 to reflect the childhood social environment while maximising sample size by including first and second generation migrants. Among all persons born in Denmark 1960, or later, we had complete reference to both parents, although data was missing for those born earlier (Pedersen et al., 2006). Immigration into Denmark was very low prior to 1960, mainly comprising migrants from other Nordic and Western European countries (Nannestad, 2004). Therefore, for ethnic density we assigned all those with missing parental data as Danish. Urbanicity was also derived at parish level based on the population density (residents per km^2^) in the year the cohort member was 15, following previous studies (Pedersen, 2001, Vassos et al., 2012).

We also linked to the Integrated Database for Longitudinal Labour Market Research (Petersson et al., 2011) deriving a parish level socio-economic index based on the proportion of residents not-employed and median gross annual income, both proxy indicators used previously (Allardyce et al., 2005, Croudace et al., 2000, Harrison et al., 2003).

### 2.6 Parental history of psychiatric disorder and socio-economic background

Parental mental health may influence the type of neighbourhood cohort members live in at age 15 and act as a confounder. Any parental psychiatric history has been associated with increased risk of psychosis (Dean et al., 2010). Therefore, we adjusted for any record of a psychiatric disorder in either parent. Parental socio-economic background may also act as a confounder (Kirkbride et al., 2014) therefore we adjusted for combined parental gross annual income at age 15, divided into quartiles within each year.

### 2.7 Exclusions – foreign born adoptees

Foreign born adoptees are at a higher risk of psychosis compared to other migrants (Cantor-Graae and Pedersen, 2013) To avoid a possible confounding effect, with adoptees more likely in low ethnic density areas, we excluded all potential adoptees (1.28%), defined as all those who were foreign born but where both (legal) parents were born in Denmark.

### 2.8 Statistical analysis

We used multilevel Poisson regression to model effects at: 1) individual 2) year (aged 15) and 3) neighbourhood (parish) levels simultaneously. The relation between ethnic density and psychosis incidence was modelled as a cross-level interaction between migrant group and neighbourhood ethnic density. The relation with urbanicity was similarly modelled as a cross-level interaction. We tested for linear trends using the Wald test.

All analyses were adjusted for age, gender (and their interaction), calendar time, and history of parental psychiatric disorder. Age and calendar time were included as time varying covariates splitting each record into age bands and time periods using the Lexis expansion method (Clayton et al., 1993). Age was categorised as: 15–20, 20–24, 25–29, 30–34, 35–39, 40–44, 45–49, 50–54, and 55 or older and calendar time into 5-year age bands, except for the 1990s where 2-year age bands were used to account for changes to the ICD system.

We also carried out the analysis using negative binomial regression, which includes an extra parameter to model over-dispersion. This made no appreciable difference and therefore only the Poisson model results are reported.

All analyses were conducted using Stata (version 14).

### 2.9 Sensitivity analysis

The ethnic density quintiles are specific to each migrant group. For example, migrants from Africa living in the lowest African density quintile (< 0.4%) are more isolated than migrants from Europe in the lowest European density quintile (< 2.3%). To test whether between group differences are therefore an artefact of this categorisation we re-ran the analysis using the following standardised categories: 1) < 1% co-ethnic density; 2) between 1 and 5% inclusive; and 3) > 5%.

### 2.10 Ethical approval

The study was approved by the Danish Data Protection Agency.

## 3 Results

### 3.1 Sample

We followed 2,224,464 people, from 1980 to 2013. During the 37,335,812 person-years of follow-up, 58,616 (2.6%) were diagnosed with a non-affective psychosis, corresponding to a crude incidence rate of 15.7 cases per 10,000 person-years at risk.

### 3.2 Incidence rates compared

For each migrant group there was an elevated incidence of non-affective psychosis (Table 1) and this was most pronounced for the African group, with a 2.93 -fold (95% CI, 2.64–3.25) increased incidence compared to Native Danes. This was least pronounced for migrants from Asia with a 1.61-fold (95% CI, 1.46–1.77) increased incidence.

**Table 1:** Incidence rates and incidence rate ratios of non-affective psychosis by migrant group.

Migrant group (country of origin)^a^	Total (N)	Person-years	Cases	Crude incidence rate^b^	Incidence rate ratio (95% CI)^c^
Denmark	1,921,874	33,804,695	24,410	7.2	1 (reference)
Africa	13,118	128,238	362	28.2	2.93 (2.64–3.25)
Europe (non-Scandinavian)	58,939	718,865	1175	16.3	1.87 (1.76–1.98)
Asia	24,512	289,412	415	14.3	1.61 (1.46–1.77)
Middle East	28,762	247,891	529	21.3	2.05 (1.88–2.23)

a Migrant group is based on the country of birth of cohort member or, if born in Denmark, country of birth of both parents.

b The incidence rate measures the number of new cases per 10,000 person years at risk.

c Incidence rate ratios were adjusted for age, gender and calendar period.

Low neighbourhood ethnic density at age 15 was associated with increased incidence of non-affective psychosis for all migrant groups (Table 2) and this effect was retained after adjusting for parental risk factors and neighbourhood urbanicity. For example, among migrants from Africa, those from areas with the lowest density of Africans had an IRR of 1.94 (95% CI, 1.17–3.23) compared to those from highest ethnic density areas and the overall linear trend appeared slightly greater than for other groups: IRR 1.22 (95% CI, 1.09–1.37). For migrants from Europe each decrease in ethnic density quintile showing a statistically significant effect while migrants from Asia and the Middle East showed a statistically significant effect for the lowest quintiles only. Parental income made a small difference and was therefore retained although adjusting for neighbourhood socio-economic profile made no statistically significant difference to model fit (*p* = 0.77) and was therefore removed. We also re-analysed the data using standardised ethnic density categories (Appendix Table 4) and our results showed a similar pattern with the African group showing the most pronounced overall ethnic density effect.

**Table 2:** Incidence rate ratios of non-affective psychosis by neighbourhood ethnic density at age 15 for each migrant group.

Ethnic density (quintiles for each group)	Ethnic density (%)	Cases	Crude Incidence Rate^a^	Incidence rate ratio (95% CI)		
				Analysis 1^b^	Analysis 2 - income adjusted^c^	Analysis 3 - urbanicity adjusted^d^
Africa						
1 (lowest)	< 0.4	60	25.8	1.79 (1.21–2.63)	1.86 (1.21–2.87)	1.94 (1.17–3.23)
2	0.4–0.9	107	37.0	2.28 (1.61–3.23)	2.06 (1.40–3.02)	2.17 (1.43–3.29)
3	0.9–1.7	67	26.0	1.39 (0.96–2.03)	1.06 (0.69–1.64)	1.11 (0.72–1.72)
4	1.7–3.7	80	26.9	1.33 (0.93–1.92)	1.17 (0.78–1.75)	1.20 (0.80–1.80)
5 (highest)	3.7–18.5	48	23.4	1	1	1
*Overall trend*^e^				*1.18 (1.09–1.28)*	*1.20 (1.10–1.32)*	*1.22 (1.09–1.37)*
Europe						
1 (lowest)	< 2.3	271	16.7	1.68 (1.37–2.06)	1.66 (1.34–2.06)	1.99 (1.56–2.54)
2	2.3–3.9	236	15.6	1.46 (1.19–1.80)	1.46 (1.17–1.81)	1.60 (1.28–1.99)
3	3.9–5.9	249	16.9	1.40 (1.14–1.72)	1.32 (1.07–1.64)	1.39 (1.12–1.72)
4	5.9–9.4	256	18.4	1.44 (1.18–1.76)	1.39 (1.13–1.71)	1.43 (1.16–1.76)
5 (highest)	9.4–26.4	163	13.8	1	1	1
*Overall trend*^e^				*1.10 (1.05–1.14)*	*1.10 (1.05–1.15)*	*1.15 (1.09–1.21)*
Asia						
1 (lowest)	< 0.6	47	15.1	1.37 (0.97–1.93)	1.53 (1.03–2.26)	1.63 (1.02–2.59)
2	0.6–1.2	64	12.7	0.98 (0.72–1.33)	0.92 (0.64–1.30)	1.00 (0.68–1.45)
3	1.2–2.1	87	13.8	0.98 (0.74–1.30)	0.87 (0.64–1.19)	0.93 (0.67–1.28)
4	2.1–3.9	97	13.4	0.91 (0.70–1.20)	0.97 (0.73–1.30)	1.00 (0.75–1.34)
5 (highest)	3.9–14.3	120	16.6	1	1	1
*Overall trend*^e^				*1.05 (0.98–1.13)*	*1.03 (0.95–1.13)*	*1.05 (0.95–1.16)*
Middle East						
1 (lowest)	< 0.8	119	22.7	1.70 (1.28–2.27)	1.45 (1.07–1.96)	1.68 (1.19–2.38)
2	0.8–1.7	106	20.7	1.30 (0.97–1.74)	1.16 (0.86–1.58)	1.29 (0.93–1.79)
3	1.7–3.3	92	18.6	1.09 (0.80–1.47)	0.98 (0.71–1.33)	1.04 (0.75–1.45)
4	3.3–6.7	107	23.1	1.28 (0.96–1.72)	1.18 (0.88–1.60)	1.23 (0.90–1.68)
5 (highest)	6.7–40.0	105	21.6	1	1	1
*Overall trend*^e^				*1.11 (1.04–1.18)*	*1.07 (1.00–1.14)*	*1.10 (1.02–1.20)*

a The incidence rate measures the number of new cases per 10,000 person years at risk.

b Adjusted for age, gender, calendar period and parental psychiatric history at age 15.

c Adjusted for age, gender, calendar period, parental psychiatric history and income at age 15.

d Adjusted for age, gender, calendar period, parental psychiatric history and income and neighbourhood urbanicity at age 15.

e Trend shows the incidence rate ratio corresponding to one quintile increase in neighbourhood ethnic density at age 15.

Looking at neighbourhood urbanicity, for native Danes (Table 3) non-affective psychosis rates increased with each increase in population density quintile, showing an overall linear trend: IRR 1.13 (95% CI, 1.11–1.14), after adjusting for parental income. Our initial analysis failed to show any statistically significant effect for migrant groups. However, after adjusting for ethnic density, while still not statistically significant between quintiles, there was an overall linear trend for migrants from Europe: IRR 1.09 (95% CI, 1.02–1.16) and the Middle East: IRR 1.12 (95% CI, 1.01–1.23); with higher rates corresponding to each increase in urbanicity quintile. For migrants from Africa and Asia we found only very weak evidence for a corresponding linear trend.

**Table 3:** Incidence rate ratios of non-affective psychosis by neighbourhood urbanicity at age 15 for each migrant group and native Danes.

Urbanicity (quintiles for each group)	Cases	Crude Incidence Rate^a^	Incidence rate ratio (95% CI)		
			Analysis 1^b^	Analysis 2 - income adjusted^c^	Analysis 3 - ethnic density adjusted^d^
Denmark					
1 (lowest)	4433	6.1	1	1	–
2	4413	6.2	1.02 (0.97–1.08)	1.07 (1.01–1.13)	–
3	4728	6.9	1.13 (1.07–1.20)	1.22 (1.15–1.29)	–
4	4893	7.6	1.21 (1.14–1.28)	1.30 (1.23–1.38)	–
5 (highest)	5943	9.7	1.56 (1.48–1.64)	1.63 (1.55–1.73)	–
*Overall trend*^e^			*1.11 (1.10–1.12)*	*1.13 (1.11–1.14)*	
Africa					
1 (lowest)	13	26.0	1	1	1
2	21	32.0	1.08 (0.54–2.16)	1.08 (0.50–2.35)	1.10 (0.50–2.39)
3	42	29.2	0.97 (0.52–1.82)	0.85 (0.42–1.74)	1.01 (0.49–2.09)
4	84	31.5	1.07 (0.59–1.92)	0.78 (0.40–1.53)	0.98 (0.48–1.99)
5 (highest)	202	26.7	0.90 (0.51–1.58)	0.73 (0.38–1.38)	1.14 (0.56–2.31)
*Overall trend*^e^			*0.93 (0.85–1.03)*	*0.90 (0.81–1.00)*	*1.03 (0.90–1.17)*
Europe					
1 (lowest)	78	18.0	1	1	1
2	72	14.5	0.80 (0.58–1.10)	0.77 (0.53–1.12)	0.76 (0.53–1.11)
3	176	17.0	0.94 (0.72–1.23)	0.92 (0.68–1.26)	1.04 (0.76–1.42)
4	321	15.0	0.79 (0.62–1.02)	0.79 (0.59–1.05)	1.05 (0.77–1.42)
5 (highest)	528	17.1	0.93 (0.73–1.18)	0.92 (0.70–1.22)	1.25 (0.93–1.69)
*Overall trend*^e^			*0.98 (0.94–1.03)*	*1.00 (0.94–1.05)*	*1.09 (1.02–1.16)*
Asia					
1 (lowest)	20	20.3	1	1	1
2	28	15.6	0.73 (0.41–1.29)	0.64 (0.32–1.32)	0.72 (0.35–1.48)
3	43	11.7	0.57 (0.34–0.97)	0.54 (0.29–1.04)	0.69 (0.35–1.35)
4	114	14.4	0.68 (0.42–1.09)	0.65 (0.36–1.16)	0.87 (0.46–1.65)
5 (highest)	210	14.4	0.66 (0.42–1.05)	0.64 (0.37–1.14)	0.87 (0.46–1.65)
*Overall trend*^e^			*0.94 (0.86–1.02)*	*0.97 (0.87–1.07)*	*1.02 (0.90–1.15)*
Middle East					
1 (lowest)	23	24.3	1	1	1
2	31	18.1	0.75 (0.43–1.28)	0.76 (0.42–1.40)	0.80 (0.43–1.47)
3	95	22.9	1.03 (0.65–1.63)	1.16 (0.70–1.94)	1.35 (0.80–2.28)
4	130	20.2	0.91 (0.58–1.43)	1.02 (0.62–1.68)	1.27 (0.76–2.14)
5 (highest)	250	21.7	0.92 (0.60–1.42)	1.12 (0.69–1.82)	1.50 (0.89–2.53)
*Overall trend*^e^			*0.98 (0.91–1.06)*	*1.04 (0.95–1.13)*	*1.12 (1.01–1.23)*

a The incidence rate measures the number of new cases per 10,000 person years at risk.

b Adjusted for age, gender, calendar period and parental psychiatric history at age 15.

c Adjusted for age, gender, calendar period, parental psychiatric history and income at age 15.

d Adjusted for age, gender, calendar period, parental psychiatric history, parental income and neighbourhood ethnic density at age 15.

e Trend shows the incidence rate ratio corresponding to one quintile increase in neighbourhood urbanicity at age 15.

To better determine how much of the increased risk could be explained by these factors we compared rates between each group and native Danes (Appendix Table 5). We found, after adjustment, that in the highest ethnic density areas the elevated risk of non-affective psychosis largely disappeared for European and Middle Eastern migrants and was much reduced for migrants from Asia and Africa.

Table 1, Table 2, Table 3 go here

## 4 Discussion

### 4.1 Summary of the results

In this nationwide study, neighbourhood ethnic density was inversely associated with incidence of non-affective psychosis for each migrant group. For some groups this appeared to mask urban/rural differences in psychosis that, when revealed, mirrored those found for the native population.

### 4.2 Strengths and limitations

This is the first study to directly examine neighbourhood effects on psychosis rates for different migrant groups with exposure determined in advance of illness onset and the first to address the joint effects of urbanicity, ethnic density and socio-economic background. The study is based on contacts to in- and out-patient psychiatric departments and visits to psychiatric emergency care units in a nation where treatment is provided through the government healthcare system free of charge, and where no private psychiatric hospitals exist. Financial factors are thus less likely to influence pathways to care in Denmark compared to many other nations (Demyttenaere et al., 2004). The population studied is representative of the Danish population as all Danish residents are included (Pedersen et al., 2006).

There are, though, some limitations to note: firstly, caution is needed when making comparisons between migrant categories as these are far from homogenous, sometimes incorporating quite disparate ethnic groups with different migration experiences. Secondly, we use the term ethnic density to refer to the density of these broad categories, in line with previous studies (Kirkbride et al., 2007b, Veling et al., 2008), while acknowledging that this remains a crude proxy for the underlying neighbourhood ethnic composition we set out to measure. It is therefore likely that effects would be even greater than those we have reported were these measures to be further refined.

### 4.3 Comparison with previous studies

The ethnic density effects in the present study are of a similar order of magnitude to those reported previously (Boydell et al., 2001, Kirkbride et al., 2014, Kirkbride et al., 2007b, Veling et al., 2008). Looking at between ethnic group differences, a recent review concludes that broadly classified Black African/Caribbean groups are most likely to be subject to an ethnic density effect as we did (Shaw et al., 2012). An advantage of our study was that we were able to investigate migrant groups otherwise overlooked in previous studies. Only one study to date has looked at this question in relation to migrants from the Middle East, covering Iraqi migrants in Sweden (*N* = 19,975), and this found no evidence for the ethnic density hypothesis (Mezuk et al., 2015). Few have looked at migrants from Europe; with these concentrating on the UK Irish population only (Cochrane and Bal, 1988, Das-Munshi et al., 2010). For the effect of urbanicity, our results for native Danes are similar to those reported previously (Pedersen, 2006, Pedersen, 2001, Pedersen and Mortensen, 2001, Vassos et al., 2012). Others have found no association for migrants (Bourque et al., 2011, Cantor-Graae and Pedersen, 2007) matching our initial results prior to adjusting for ethnic density.

### 4.4 Interpretation

We were able to show clear and consistent ethnic density effects for different migrant groups which may be, in part, because we could access whole population data with exposure determined prior to the outcome of interest. It is also possible that this may partly reflect a greater isolation of migrants in Denmark, as others have suggested (Valentine et al., 2009). The urbanicity results fit the ‘ethnic density/protection’ interpretation proposed to explain the apparent absence of urban-rural differences for migrant groups (Cantor-Graae and Pedersen, 2007). That this did not apply to migrants from Africa may well be because of their much lower representation outside of the most urban areas (Appendix Table 6). For migrants from Asia the ethnic density effect was weakest, and only applied to the lowest quintile, which may explain why adjusting for this made little difference.

As we have shown, the overall contribution of these neighbourhood factors can explain much of the increased risk of psychosis for some migrant groups. Each factor is, of course, itself likely to be a proxy for some underlying mechanism, with a possible key determinant being exposure to a socially stressful environment (Cantor-Graae et al., 2003, Lederbogen et al., 2013)***.*** There is some evidence that living in a higher ethnic density area may reduce social stress through improved social support and access to social capital which, in turn, can act as a buffer against discrimination (Becares and Das-Munshi, 2013, Das-Munshi et al., 2010, Kirkbride et al., 2007b)***.***

### 4.5 Conclusion

To conclude, our findings show, using the most rigorous study design to date, clear ethnic density effects related to psychosis incidence. We also demonstrate how neighbourhood urbanicity is a relevant factor for some migrant groups, but only when ethnic density is accounted for. The effects demonstrated are on a scale that suggests the neighbourhood environment is a clinically important factor influencing psychosis risk.

## Contributors

PS was responsible for the initial study design, analysis and interpretation of the study and drafted the manuscript. LB, JD, EA, CP and MT were all involved in study design and interpretation of results. All authors contributed to and have approved the final manuscript.

## Conflict of interest

The study authors have nothing to disclose.

## Funding

This work was supported by a UK Medical Research Council Fellowship (MR/K021494/1) to P.S.; Hallsworth Research Fellowship and a UK Economic and Social Research Council Future Research Leaders grant (ES/K001582/1) to L.B.; Aarhus University, the Lundbeck Foundation, the Stanley Medical Research Institute to both E.A. and C.P.; and the Health Foundation working with the Academy of Medical Sciences to J.D.

## Appendix A Supplementary data

Supplementary material. Image 1
