# Supplementary material for: Ethnic density, urbanicity and psychosis risk for migrant groups – A population cohort study
Source: Schizophr Res. 2017 Dec;190:82–7. doi: 10.1016/j.schres.2017.03.032 (PMC5735221; doi:10.1016/j.schres.2017.03.032)
Supplement: Supplementary file 1 — Supplementary material. [file mmc1.doc]

## Appendix

Table 4 Incidence rate ratios of non-affective psychosis by neighbourhood ethnic density at age 15 (standardised categories) for each migrant group

|  |  |  |  | Incidence rate ratio (95% CI) | | |
| --- | --- | --- | --- | --- | --- | --- |
| Ethnic density  (for each migrant group) | Ethnic density (%) | Cases | Crude Incidence  Rate^a^ | Analysis 1^b^ | Analysis 2  (adjusted for parental income)^c^ | Analysis 3  (further adjusted for urbanicity)^d^ |
| Africa |  |  |  |  |  |  |
| 1 (lowest) | <1.0 | 178 | 31.6 | 3.62 (2.15 - 6.07) | 2.96 (1.73 - 5.09) | 3.05 (1.73 - 5.38) |
| 2 | 1.0 - 5.0 | 168 | 27.7 | 2.33 (1.39 - 3.92) | 1.75 (1.02 - 3.00) | 1.79 (1.04 - 3.09) |
| 3 (highest) | >5.0 | 16 | 14.2 | 1 | 1.00 (1.00 - 1.00) | 1.00 (1.00 - 1.00) |
| Europe |  |  |  |  |  |  |
| 1 (lowest) | <1.0 | 47 | 13.0 | 1.31 (0.97 - 1.77) | 1.17 (0.80 - 1.70) | 1.25 (0.84 - 1.88) |
| 2 | 1.0 - 5.0 | 608 | 16.6 | 1.27 (1.12 - 1.43) | 1.26 (1.11 - 1.44) | 1.31 (1.14 - 1.50) |
| 3 (highest) | >5.0 | 520 | 16.5 | 1 | 1.00 (1.00 - 1.00) | 1.00 (1.00 - 1.00) |
| Asia |  |  | 0.0 |  |  |  |
| 1 (lowest) | <1.0 | 96 | 14.1 | 1.49 (1.08 - 2.06) | 1.52 (1.06 - 2.17) | 1.57 (1.05 - 2.35) |
| 2 | 1.0 - 5.0 | 252 | 14.2 | 1.16 (0.88 - 1.53) | 1.21 (0.90 - 1.63) | 1.25 (0.92 - 1.69) |
| 3 (highest) | >5.0 | 67 | 15.3 | 1 | 1.00 (1.00 - 1.00) | 1.00 (1.00 - 1.00) |
| Middle East |  |  |  |  |  |  |
| 1 (lowest) | <1.0 | 145 | 22.0 | 1.49 (1.17 - 1.91) | 1.28 (0.99 - 1.66) | 1.38 (1.02 - 1.86) |
| 2 | 1.0 - 5.0 | 238 | 20.7 | 1.14 (0.91 - 1.42) | 1.03 (0.82 - 1.29) | 1.05 (0.82 - 1.34) |
| 3 (highest) | >5.0 | 146 | 21.8 | 1 | 1.00 (1.00 - 1.00) | 1.00 (1.00 - 1.00) |

^a^The incidence rate measures the number of new cases per 10,000 person years at risk  ^b^Adjusted for age, gender, calendar period and parental psychiatric history at age 15
^c^Adjusted for age, gender, calendar period, parental psychiatric history and income at age 15
^d^Adjusted for age, gender, calendar period, parental psychiatric history and income and neighbourhood urbanicity at age 15

Table 5 Incidence rate ratios of non-affective psychosis by neighbourhood ethnic density at age 15 for each migrant group - compared with native Danes in lowest ethnic density quintile

|  |  |  |  | Incidence rate ratio (95% CI) | | |
| --- | --- | --- | --- | --- | --- | --- |
| Ethnic density (for each migrant group) | Ethnic density (%) | Cases | Crude Incidence  Rate^a^ | Analysis 1^b^ | Analysis 2  (adjusted for parental income)^c^ | Analysis 3  (further adjusted for urbanicity)^d^ |
| Africa |  |  |  |  |  |  |
| 1 (lowest) | <0.4 | 60 | 25.8 | 3.14 (2.44 - 4.05) | 2.58 (1.92 - 3.46) | 2.88 (1.54 - 5.40) |
| 2 | 0.4 - 0.9 | 107 | 37.0 | 4.02 (3.32 - 4.87) | 2.84 (2.27 - 3.56) | 3.22 (1.61 - 6.43) |
| 3 | 0.9 - 1.7 | 67 | 26.0 | 2.45 (1.92 - 3.12) | 1.47 (1.10 - 1.98) | 1.64 (0.78 - 3.45) |
| 4 | 1.7 - 3.7 | 80 | 26.9 | 2.35 (1.87 - 2.95) | 1.62 (1.25 - 2.09) | 1.78 (0.85 - 3.71) |
| 5 (highest) | 3.7 - 18.5 | 48 | 23.4 | 1.76 (1.31 - 2.36) | 1.38 (1.01 - 1.90) | 1.48 (0.69 - 3.20) |
| Europe |  |  |  |  |  |  |
| 1 (lowest) | <2.3 | 271 | 16.7 | 2.25 (2.00 - 2.54) | 1.83 (1.60 - 2.10) | 2.08 (1.59 - 2.72) |
| 2 | 2.3 - 3.9 | 236 | 15.6 | 1.96 (1.72 - 2.23) | 1.61 (1.40 - 1.85) | 1.67 (1.23 - 2.25) |
| 3 | 3.9 - 5.9 | 249 | 16.9 | 1.88 (1.65 - 2.14) | 1.46 (1.27 - 1.68) | 1.45 (1.06 - 1.99) |
| 4 | 5.9 - 9.4 | 256 | 18.4 | 1.93 (1.70 - 2.20) | 1.53 (1.33 - 1.76) | 1.49 (1.09 - 2.04) |
| 5 (highest) | 9.4 - 26.4 | 163 | 13.8 | 1.34 (1.13 - 1.58) | 1.10 (0.93 - 1.31) | 1.04 (0.75 - 1.46) |
| Asia |  |  |  |  |  |  |
| 1 (lowest) | <0.6 | 47 | 15.1 | 2.22 (1.67 - 2.96) | 1.89 (1.35 - 2.65) | 2.64 (1.50 - 4.66) |
| 2 | 0.6 - 1.2 | 64 | 12.7 | 1.58 (1.24 - 2.03) | 1.13 (0.85 - 1.52) | 1.61 (0.86 - 3.03) |
| 3 | 1.2 - 2.1 | 87 | 13.8 | 1.60 (1.29 - 1.98) | 1.08 (0.84 - 1.38) | 1.50 (0.78 - 2.89) |
| 4 | 2.1 - 3.9 | 97 | 13.4 | 1.48 (1.21 - 1.82) | 1.21 (0.97 - 1.50) | 1.62 (0.84 - 3.13) |
| 5 (highest) | 3.9 - 14.3 | 120 | 16.6 | 1.62 (1.34 - 1.96) | 1.24 (1.01 - 1.52) | 1.62 (0.84 - 3.13) |
| Middle East |  |  |  |  |  |  |
| 1 (lowest) | <0.8 | 119 | 22.7 | 1.98 (1.65 - 2.37) | 1.36 (1.11 - 1.66) | 1.38 (0.86 - 2.21) |
| 2 | 0.8 - 1.7 | 106 | 20.7 | 1.51 (1.24 - 1.83) | 1.09 (0.89 - 1.34) | 1.06 (0.63 - 1.77) |
| 3 | 1.7 - 3.3 | 92 | 18.6 | 1.26 (1.02 - 1.55) | 0.91 (0.73 - 1.14) | 0.85 (0.50 - 1.46) |
| 4 | 3.3 - 6.7 | 107 | 23.1 | 1.49 (1.22 - 1.82) | 1.11 (0.90 - 1.36) | 1.01 (0.59 - 1.73) |
| 5 (highest) | 6.7 - 40.0 | 105 | 21.6 | 1.16 (0.93 - 1.45) | 0.94 (0.74 - 1.18) | 0.82 (0.47 - 1.44) |

^a^The incidence rate measures the number of new cases per 10,000 person years at risk ^b^Adjusted for age, gender, calendar period and parental psychiatric history at age 15
^c^Adjusted for age, gender, calendar period, parental psychiatric history and income at age 15
^d^Adjusted for age, gender, calendar period, parental psychiatric history and income and neighbourhood urbanicity at age 15

Table 6 Sample size for each combination of neighbourhood ethnic density and
urbanicity category by migrant group

|  | Ethnic density | | | | |  | |  | |
| --- | --- | --- | --- | --- | --- | --- | --- | --- | --- |
| Urbanicity | 1 (low) | 2 | 3 | 4 | 5 (High) | |  | | *Total* |
|  |  |  |  |  |  | |  | |  |
| Africa |  |  |  |  |  | |  | |  |
| 1 (lowest) | 343 | 110 | 12 | 12 | 0 | |  | | *477* |
| 2 | 385 | 223 | 65 | 9 | 0 | |  | | *682* |
| 3 | 420 | 504 | 366 | 214 | 56 | |  | | *1560* |
| 4 | 381 | 859 | 686 | 660 | 57 | |  | | *2643* |
| 5 (highest) | 220 | 875 | 1,471 | 2,226 | 2,964 | |  | | *7756* |
|  |  |  |  |  |  | |  | |  |
| *Total* | *1749* | *2571* | *2,600* | *3,121* | *3,077* | |  | | *13,118* |
|  |  |  |  |  |  | |  | |  |
| Europe |  |  |  |  |  | |  | |  |
| 1 (lowest) | 2,070 | 1,005 | 534 | 456 | 101 | |  | | *4,166* |
| 2 | 2,613 | 942 | 336 | 226 | 221 | |  | | *4,338* |
| 3 | 2,633 | 2,463 | 1,814 | 1,123 | 334 | |  | | *8,367* |
| 4 | 1,795 | 3,142 | 3,460 | 4,160 | 4,999 | |  | | *17,556* |
| 5 (highest) | 1,047 | 3,385 | 5,738 | 6,694 | 7,648 | |  | | *24,512* |
|  |  |  |  |  |  | |  | |  |
| *Total* | *10,158* | *10,937* | *11,882* | *12,659* | *13,303* | |  | | *58,939* |
|  |  |  |  |  |  | |  | |  |
| Asia |  |  |  |  |  | |  | |  |
| 1 (lowest) | 486 | 368 | 82 | 12 | 115 | |  | | *1,063* |
| 2 | 501 | 653 | 455 | 174 | 1 | |  | | *1,784* |
| 3 | 383 | 925 | 1,059 | 704 | 216 | |  | | *3,287* |
| 4 | 281 | 828 | 1,540 | 2,109 | 1,849 | |  | | *6,607* |
| 5 (highest) | 213 | 957 | 2,045 | 3,390 | 5,166 | |  | | *11,771* |
|  |  |  |  |  |  | |  | |  |
| *Total* | *1864* | *3731* | *5,181* | *6,389* | *7,347* | |  | | *24,512* |
|  | |  |  |  |  | |  | |  |
| Middle East | |  |  |  |  | |  | |  |
| 1 (lowest) | 716 | 245 | 52 | 0 | 0 | |  | | *1,013* |
| 2 | 1,057 | 740 | 151 | 5 | 0 | |  | | *1,953* |
| 3 | 1,073 | 1,346 | 922 | 1,155 | 140 | |  | | *4,636* |
| 4 | 849 | 1,731 | 2,317 | 1,973 | 365 | |  | | *7,235* |
| 5 (highest) | 742 | 1,363 | 2,535 | 3,016 | 6,269 | |  | | *13,925* |
|  |  |  |  |  |  | |  | |  |
| *Total* | *4437* | *5425* | *5977* | *6149* | *6774* | |  | | *28,762* |
